# Hemispheric biases and the control of visuospatial attention: an ERP study

**DOI:** 10.1186/1471-2202-6-51

**Published:** 2005-08-24

**Authors:** Kevin M Spencer, Marie T Banich

**Affiliations:** 1 Department of Psychiatry, Harvard Medical School/VA Boston Healthcare System, Brockton, Massachusetts USA; 2 Department of Psychology, University of Colorado at Boulder, Boulder, Colorado USA

## Abstract

**Background:**

We examined whether individual differences in hemispheric utilization can interact with the intrinsic attentional biases of the cerebral hemispheres. Evidence suggests that the hemispheres have competing biases to direct attention contralaterally, with the left hemisphere (LH) having a stronger bias than the right hemisphere. There is also evidence that individuals have characteristic biases to utilize one hemisphere more than the other for processing information, which can induce a bias to direct attention to contralateral space. We predicted that LH-biased individuals would display a strong rightward attentional bias, which would create difficulty in selectively attending to target stimuli in the left visual field (LVF) as compared to right in the performance of a bilateral flanker task.

**Results:**

Consistent with our hypothesis, flanker interference effects were found on the N2c event-related brain potential and error rate for LH-biased individuals in the Attend-LVF condition. The error rate effect was correlated with the degree of hemispheric utilization bias for the LH-Bias group.

**Conclusion:**

We conclude that hemispheric utilization bias can enhance a hemisphere's contralateral attentional bias, at least for individuals with a LH utilization bias. Hemispheric utilization bias may play an important and largely unrecognized role in visuospatial attention.

## Background

In this study we investigated the hypothesis that the control of spatial attention is influenced by *hemispheric utilization bias*, a characteristic bias of individuals to utilize one hemisphere more than the other for information processing [1]. Spatial selective attention is commonly thought to be mediated by a network in which attentional control systems based in prefrontal and posterior parietal areas modulate processing in perceptual areas [2,3]. Each of the cerebral hemispheres contains a set of mechanisms for attentional control and perceptual representation [4,5], which may be capable of operating independently in the intact brain [6]. These hemispheric attention systems appear to have mutually inhibitory biases that compete to direct attention to contralateral space, with the left hemisphere (LH) having a stronger and/or more focused intrinsic bias than the right hemisphere (RH) [7].

Evidence for interhemispheric attentional competition comes from studies showing asymmetrical gradients in spatial attention in healthy individuals [8,9], split-brain patients [10-12], and neglect patients [13,14]. Also in neglect patients, disruption of the contralesional hemisphere by a subsequent stroke [15] or transcranial magnetic stimulation (TMS) [16] can cause recovery from neglect symptoms, consistent with the lesioned hemisphere being released from inhibition by the contralesional hemisphere. Furthermore, in healthy individuals, reduction of the excitability of the parietal cortex in one hemisphere by TMS can lead to an increase in subjects' ability to attend to ipsilateral stimuli [17]. Together, these lines of evidence point to the focus of spatial attention being influenced by mutually competitive hemispheric attention systems.

Hemispheric utilization bias appears to represent another, largely unexplored type of interhemispheric competition. Individuals appear to have a characteristic and consistent bias to utilize one hemisphere more than the other for processing information. A reliable finding from many studies addressing the issue of between-subject variance on laterality tasks has been that the major source of this variability is not random measurement error nor variability in hemispheric specialization. Rather, a significant source of variation comes from characteristic biases that individuals possess for consistently utilizing one hemisphere more than the other for processing information [1,18-22].

For example, Levine and colleagues [21] tested individuals in three perceptual recognition tasks with bilateral stimulus presentations using words, faces, and pictures of chairs. As a group, the participants demonstrated a right visual field (RVF)/LH advantage on the word task, a left visual field (LVF)/RH advantage on the face task, and no visual field advantage on the chair task, as was expected. Yet individuals differed with respect to the size and direction of these advantages. Levine et al. found that individuals' asymmetry scores on the tasks were correlated, such that participants with a RVF/LH advantage on the chair task had a larger-than-average RVF/LH advantage on the word task, and a smaller-than-average LVF/RH advantage on the face task. Hence, across all tasks these individuals were biased to utilize their LH more than average across all tasks. The reverse pattern was found for participants with a LVF/RH advantage on the chair task. The common factor affecting a given individual's performance on the three tasks is what we call here *hemispheric utilization bias *(referred to as "characteristic perceptual asymmetry" by some researchers). When the inter-subject variability due to hemispheric utilization bias is taken into account, the pattern of hemispheric specialization across a population of individuals is consistent with that predicted by studies of neurological patients [23]. Thus, hemispheric utilization bias is stable within an individual but variable across individuals.

Levy and colleagues [1] proposed that hemispheric utilization bias induces a characteristic bias to direct attention to the side of space contralateral to the more activated hemisphere, which leads to enhanced processing of information in the contralateral field regardless of the nature of the stimulus. Thus, it seems plausible that individuals' hemispheric utilization biases could interact with the intrinsic attentional biases of the hemispheres.

**Figure 1:**
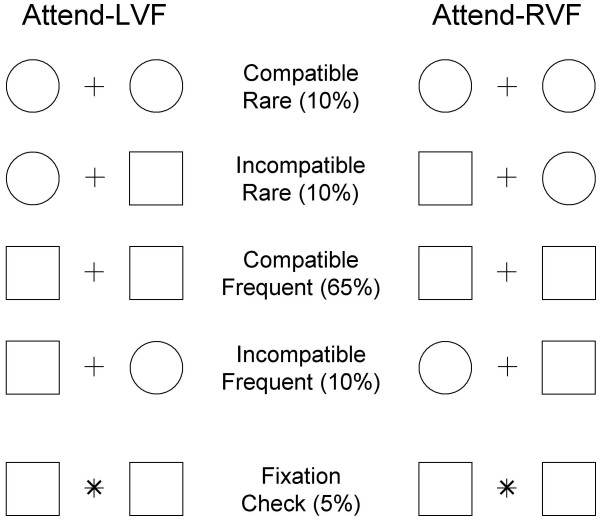
**Stimulus arrays**. The stimulus arrays in each attention condition are shown, along with the percentage of trials on which they were presented.

In the present study we investigated this hypothesis, predicting that the intrinsic attentional bias of a hemisphere to the contralateral side of space would be enhanced when an individual is also biased to utilize that same hemisphere. Competition between the hemispheres in attentional control was assessed using a bilateral version of the Eriksen flankers task [24], in which participants responded to target stimuli in the attended hemifield while ignoring a simultaneous flanking distractor in the opposite hemifield (Figure 1). Flanker items could be Compatible (same response as target) or Incompatible (opposite response). The attended hemifield was varied across blocks.

We expected that the effect of the flanker would be larger when presented to the hemisphere for which an individual has a utilization bias, compared to when the it was presented to the opposite hemisphere. Furthermore, this effect would be modulated by the greater strength of the LH's intrinsic bias compared to the RH. In particular, we predicted that LH-biased individuals would show the largest flanker effects when they attended to target items in the left visual field (LVF) and tried to ignore flankers in the right visual field (RVF), for which they would have a strong attentional bias. Hemispheric utilization bias was measured using the chimeric faces test (CFT; [25]) (see Methods).

Studies of neglect and extinction patients have found evidence that stimuli presented to each hemisphere are processed to a post-identification level without reaching awareness, suggesting that interhemispheric attentional competition occurs at a late stage of processing [26-29]. Therefore, we focused the present investigation on the N2c component of the ERP, rather than on early sensory components such as the P1 and N1 that reflect the spatial distribution of early selective attention processes [30]. The N2c is elicited by stimuli that are associated with response conflict, such as a "no-go" stimulus in a "go/no-go" task [31], or by stimulus displays containing response-incompatible distractors, as in flanker [32,33] or Stroop tasks [34]. N2c amplitude is related to the degree of response conflict on correct-response trials [32], even when no overt signs of response conflict are present, suggesting that this component may reflect both the detection and suppression of incorrect response information [35]. The N2c is maximal at midline fronto-central sites with a peak around 200–300 ms post-stimulus in tasks with simple stimuli. Source localization studies suggest that the N2c may be generated in the anterior cingulate cortex, similar to the error related negativity [33,34].

## Results

**Figure 2:**
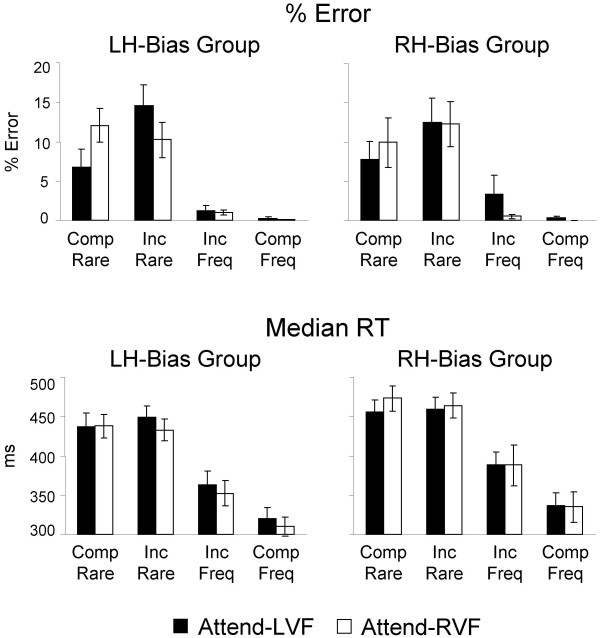
**Behavioral performance data**. Error rate and median RT data for each hemispheric utilization bias group. Bars indicate standard error. Comp = Compatible, Inc = Incompatible.

**Figure 3:**
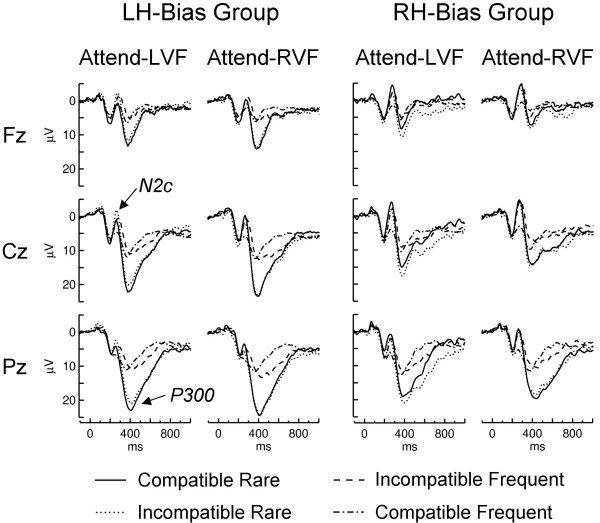
**Grand average ERPs (whole epoch)**. ERPs in each condition at electrodes Fz, Cz, and Pz are shown for the LH-Bias and RH-Bias groups. The N2c and P300 components are indicated.

**Figure 4:**
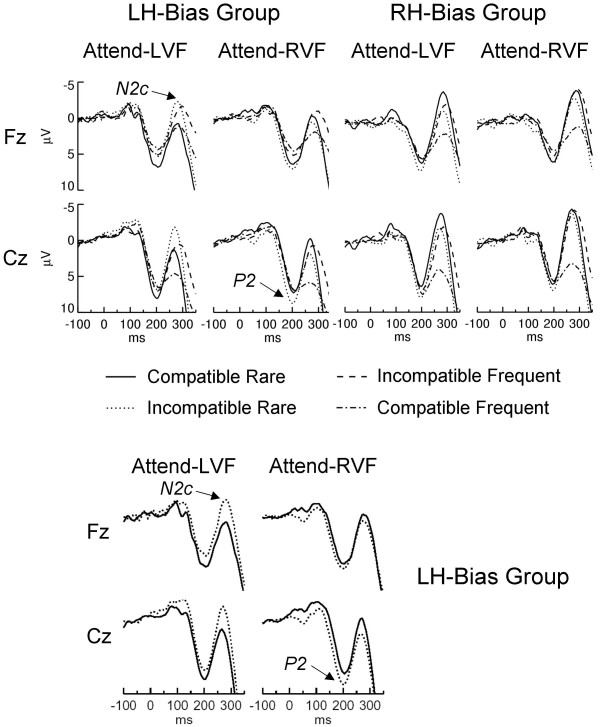
**Grand average ERPs (early portion of epoch)**. ERPs in the early portion of the epoch at Fz and Cz are shown to highlight the N2c and P2 effects. In the bottom panel, only the Rare stimulus ERPs are shown for the LH-Bias Group.

The behavioral data are presented in Fig. 2, and the ERP data in Figs. 3 and 4. As is typical in an oddball paradigm, rare stimuli compared to frequents were associated with higher error rates, slower reaction times (RTs), and elicited larger N2 and P300 components (see Fig. 3), so these effects will not be discussed further. We focused our analyses on the responses elicited by Compatible and Incompatible Rares, which are matched for stimulus probability.

As shown in Fig. 2, Incompatible Rares elicited more errors than Compatible Rares (Flanker main effect: *F*[1,22] = 15.8, *p *< 0.001). This difference was larger in the Attend-LVF condition than in Attend-RVF (*F*[1,22] = 12.7, *p *< 0.01), and the Attention × Flanker interaction was statistically significant for the LH-Bias group but not the RH-Bias group (LH-Bias: *F*[1,11] = 19.8, *p *< 0.001; RH-Bias: *F*[1,11] = 0.861, n.s.; Group × Attention × Flanker: *F*[1,22] = 4.57, *p *< 0.05). Planned comparisons confirmed these effects. Thus, LH-biased individuals were more likely to make an error on rare trials when a response-incompatible flanker was presented to their biased hemisphere. In the RT data, the overall Attention × Flanker interaction approached significance (*F*[1,22] = 3.99, *p *= 0.058), but the Group × Attention × Flanker interaction was not significant (*F*[1,22] = 0.177, n.s.).

As predicted, Incompatible Rare stimuli elicited a larger N2c component (245–325 ms) than Compatible Rares only in the Attend-LVF condition for the LH-Bias group (Group × Attention × Flanker: *F*[1,22] = 8.86, *p *< 0.01; LH-Bias, Attention × Flanker: *F*[1,11] = 13.7, *p *< 0.01; RH-Bias, Attention × Flanker: *F*[1,11] = 0.695, n.s.). Direct comparison of the responses for the LH-Bias group confirmed that Incompatible Rares elicited larger N2s than Compatible Rares in Attend-LVF (*F*[1,11] = 9.22, *p *< 0.05), but there was no difference in Attend-RVF (*F*[1,11] = 2.79, n.s.). For the RH-Bias group there was no interaction between attention and flanker compatibility (Attention × Flanker: *F*[1,11] = 0.695, n.s.; Group × Attention × Flanker: *F*[1,22] = 8.86, *p *< 0.01).

Inspection of the waveforms in Fig. 4 suggests that the P2 component (160–240 ms), which peaks before the N2c, shows a complementary pattern of differences between Compatible and Incompatible Rares across the two attention conditions: the P2 is increased rather than decreased in amplitude for Compatible Rares compared to Incompatible Rares. For the P2, the Group × Attention × Flanker interaction approached significance (*F*[1,22] = 3.75, *p *= 0.066), and the Attention × Flanker interaction was significant for the LH-Bias group (*F*[1,11] = 10.52, *p *< 0.01) but not the RH-Bias group (*F*[1,11] = 0.304, n.s.). For the LH-Bias group, there was a trend for P2 amplitude to be larger for Compatible relative to Incompatible Rares in Attend-LVF (*F*[1,11] = 3.50, *p *= 0.088), and P2s elicited by Incompatible Rares were significantly larger than Compatible Rares in Attend-RVF (*F*[1,11] = 5.25, *p *< 0.05). The marginal P2 effect in the Attend-LVF condition may reflect overlap with the N2c, especially given the similar topography of the effects (maximal at Fz), but the P2 effect in Attend-RVF is harder to interpret. The P2 is seldom reported to be modulated by spatial attention, and to our knowledge, has not been reported to be modulated by response compatibility. At this point, we can only speculate that the Attend-RVF P2 effect could be due to a registration of the incompatible flanker at a perceptual, rather than response-related level.

Since we found relationships between hemispheric utilization bias and some of the dependent variables, we investigated whether individual differences in these measures might be related. Non-parametric Spearman's correlation coefficients (2-tailed) were computed between CFT score, the Attend-LVF error rate effect (Incompatible Rare minus Compatible Rare), and the Attend-LVF N2c effect (Incompatible Rare minus Compatible Rare) separately for each group. For the LH-Bias group, CFT score and the error rate effect were significantly correlated (ρ = 0.591, *p *< 0.05), indicating that the greater the degree of LH bias, the more the individuals were influenced by conflicting items presented to their LH. For the RH-Bias group, a negative correlation between CFT score and error rate approached significance (ρ = -0.515, *p *= 0.087). No other correlations with CFT score were found.

## Discussion

When attending to the LVF, the LH-Bias group made more errors when the flanker presented to their biased hemisphere conflicted with the target item in the opposite visual field. The size of this effect was correlated with the degree of LH utilization bias as measured by the CFT. Furthermore, on correct trials, Incompatible Rare arrays elicited larger N2c components than Compatible Rare arrays, indicating that the conflict between the flanker and target reached a late, response-related stage of processing. Taken together, these data suggest that when LH-biased individuals focus attention in the LVF, they still attend at some level to items in the task-irrelevant RVF because of their hemispheric utilization bias.

Flanker compatibility had a much stronger effect on error rate than RT for the Rare arrays. The most likely explanation for this discrepancy is that the slowing of RT due to the low probability of the Rares overrode the effects of flanker compatibility. In unpublished data, we have obtained flanker effects on RT using the same displays but with equal stimulus probabilities [36]. Another possibility is that the manner in which individuals allocated attention between the visual fields varied across trials. On the majority of Incompatible Rare trials, the potency of the RVF flankers influenced processing (manifested by the N2c) but ultimately not overt response production (no effect on RT). However, on a sizable proportion of Incompatible Rare trials, the influence of the conflicting RVF flankers did reach the level of response production, leading to errors.

These data demonstrate that interhemispheric competition for the control of spatial attention can indeed be influenced by individual differences in hemispheric utilization. We predicted that when individuals have a LH utilization bias, it would augment the LH's already strong intrinsic attentional bias to the contralateral visual field. This enhancement of the LH's attentional bias would allow task-irrelevant information presented in the RVF to interfere with task-relevant LVF items. For the RH-Bias group, however, no effects of interhemispheric attentional competition were detected for the corresponding set of conditions (incompatible LVF flankers in the Attend-RVF condition). This outcome may be due to the weaker intrinsic attentional bias of the RH to the contralateral side of space. Further research is necessary to examine whether hemispheric utilization bias can influence attentional control by the RH.

The N2c data are consistent with the existence of a late locus of interhemispheric attentional competition, as demonstrated by behavioral studies [26-29]. The N2c results could also be interpreted in terms of the LH attempting to control response output, rather than as a failure to control spatial attention. Such a proposal would be consistent with the evidence from split-brain patients for an interhemispheric "bottleneck" in response control [37,38].

An important question to be examined in future studies is whether hemispheric utilization bias influences the early stages of spatial selective attention that are manifested by the P1 and posterior N1 components [30]. The present task was not well-suited to examine these early attention effects due to the bilateral stimulus displays, relatively small number of stimuli, and long stimulus onset asynchrony (SOA). The P1 and N1 attention effects are typically obtained in tasks involving unilateral displays (but see [39]) and hundreds of trials with rapid stimulus presentation (~300 ms SOA). SOA may be a particularly important factor, as we have found in unpublished data that as SOA decreases, flanker compatibility effects also decrease. Nevertheless, the examination of early spatial attention effects will be necessary to fully determine the mechanisms by which hemispheric attentional biases operate.

## Conclusion

This study provides evidence that hemispheric utilization bias influences visuospatial attention at a late, response-related stage of processing, by enhancing a hemisphere's intrinsic contralateral attentional bias. These data are consistent with the hypothesis of Kinsbourne [7] that the cerebral hemispheres have mutually inhibitory and asymmetric biases to direct attention to contralateral space. The results of this study suggest that hemispheric utilization bias may play an important and largely unrecognized role in visuospatial attention.

## Methods

### Chimeric faces test (CFT)

**Figure 5:**
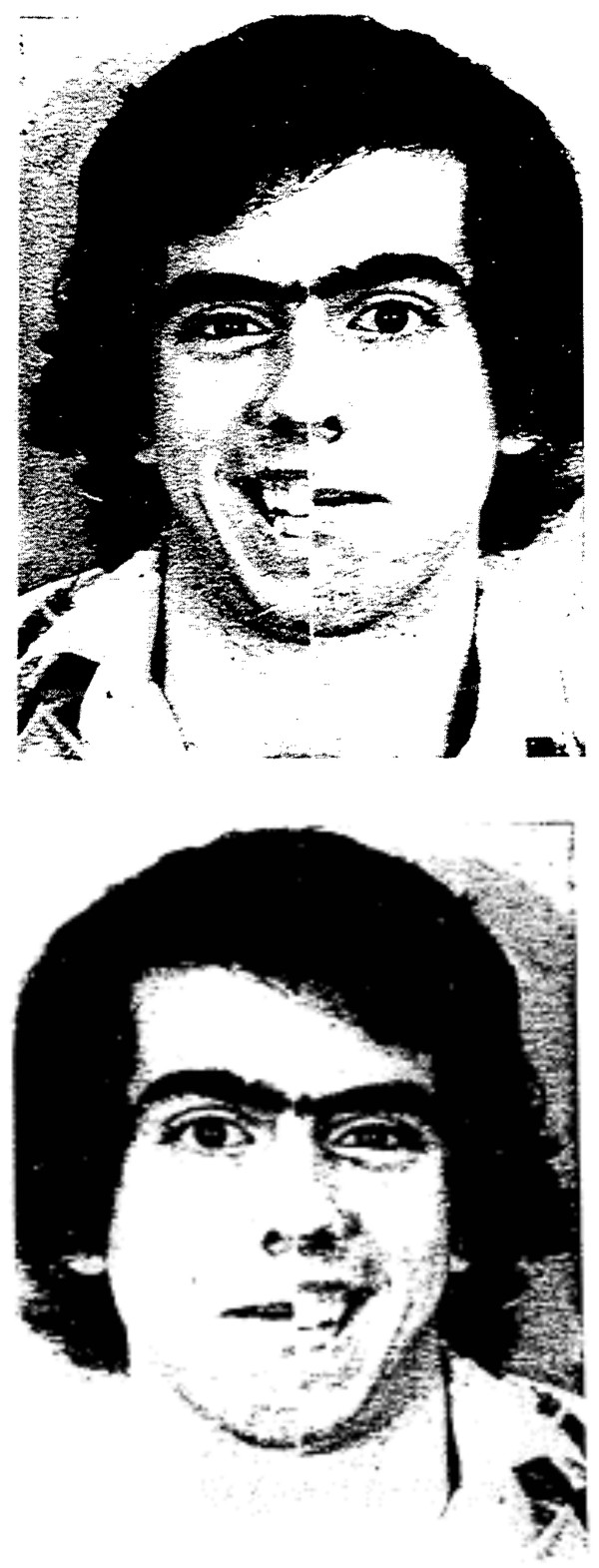
**Chimeric Faces Test (CFT)**. A sample page from the CFT (see Methods), which was used to measure hemispheric utilization bias.

On each trial of the CFT [25], participants are shown two chimeric faces, each composed of a smiling half-face and a neutral half-face, which are mirror images of one another (Fig. 5). Participants are given several seconds to scan the faces in free vision, and their task is to decide which of the two faces appears happier than the other. The version of the CFT used here was administered in booklet form, with responses marked on a response sheet. The CFT score is computed according to the formula (R - L)/(R + L), where R and L are the total number of times an individual chooses a face with a right or left smile. Hence, the CFT score indicates the degree and direction of an individual's perceptual asymmetry. It ranges from -1.0 (complete leftward bias) to +1.0 (complete rightward bias).

Across individuals there is a left hemispatial bias on the CFT, in agreement with a RH advantage for processing faces and facial expressions in particular [40]. It is the degree and direction in which an individual differs from this mean that reflects hemispheric utilization bias. Some of the features of this test that make it a useful measure of hemispheric utilization bias are its high test-retest and split-half reliability [25] and its sensitivity to attentional biases, such as those seen in neglect [41].

### Participants

Participants were recruited from the university community and received monetary reimbursement. All gave their informed consent to participate in the study, as per the guidelines of the Institutional Review Board of the University of Illinois at Urbana-Champaign and in accordance with the Helsinki Declaration. Participants were right-handed as assessed by the Edinburgh inventory [42] and had normal or corrected-to-normal vision.

Twenty-seven individuals participated in the study. The data from 3 were unusable due to data acquisition problems, resulting in a final sample of 24 (13 females, 11 males; age range 18–32 years). They were divided into two groups (N = 12 each) based on their performance on the CFT. The LH-Bias group comprised all individuals with a score indicating more LH involvement than the mean (-0.377, an estimate of the population mean based on a sample of 1000 individuals; Heller, unpublished data, 1996), and the RH-Bias group comprised all individuals with a score indicating more RH involvement than the mean. The overall mean CFT score was -0.202, which did not significantly differ from the population estimate (*t*[23] = 1.56, *p *= 0.132). The LH-Bias group consisted of 7 females and 5 males, with a mean CFT score of +0.262. The RH-Bias group consisted of 6 females and 6 males, with a mean CFT score of -0.667. By subtracting the population mean CFT score from each individual's score, we found that the absolute degree of hemispheric utilization bias was larger for the LH-Bias group than the RH-Bias group (*t*[22] = 3.054, *p *< 0.01).

### Stimuli and task design

Stimulus arrays (Fig. 1) consisted of two items placed to the left and right of the center of the display on the horizontal meridian. Stimulus items were line drawings of circles and squares presented in white on a black background. All stimulus elements were 2° wide, with their inner edges 4° from the center of the display. A fixation cross was continuously present at the center of the display. The stimulus arrays were presented for 150 ms in white on a black background, with a 2000 ms SOA.

After giving informed consent, participants filled out medical history and handedness questionnaires and took the CFT. Following electrode application, they were seated in a darkened sound-attenuation booth and rested their head on a chinrest in front of the computer monitor on which the stimuli were presented.

Participants performed a bilateral version of the Eriksen flanker task [24] in an "oddball" format. They responded to target stimuli in one hemifield while ignoring a simultaneous flanking distractor in the opposite hemifield (Fig. 1). The flanker could be Compatible (same stimulus as the target) or Incompatible (the other potential target item). To increase the potential for response conflict, the stimulus arrays were presented with different probabilities. Arrays with circle targets were rare (20%) and arrays with square targets were frequent (80%). Thus, stimulus arrays were Compatible Rare (circle for target and flanker), Incompatible Rare (circle target, square flanker), Compatible Frequent (square for target and flanker), and Incompatible Frequent (square target, circle flanker). The different stimulus probabilities ensured that participants would develop a bias to make a "square" response, so that response conflict would be maximal on trials in which the target item was a circle and the flanker was a square (Incompatible Rares).

The flanker task was performed while attending to the LVF and RVF (Attend-LVF and Attend-RVF conditions). The task was to press one button for circles and another for squares in the attended visual field using separate fingers on the same hand, responding as quickly and accurately as possible. Each attention condition was performed twice, once with each hand. Practice trials were given prior to each attention condition to familiarize the participants with task requirements.

Participants were instructed to maintain central fixation, which was monitored by the horizontal electro-oculogram (EOG) (see below). They were also instructed to make verbal responses (saying the word "check") to infrequent fixation check stimuli. On fixation check trials (5% of the total trials), an "X" (1° wide) was presented at the center of the display, overlapping the fixation cross. The fixation check stimuli were presented simultaneously with Compatible Frequent arrays. No participant missed more than 1 or 2 fixation check stimuli.

The order of the attention conditions and response hands was counterbalanced across participants. In each combination of attention condition and response hand, participants received 24 trials (10%) each of Compatible Rare, Incompatible Rare, and Incompatible Frequent arrays; 156 (65%) Compatible Frequent arrays; and 12 (5%) Fixation Check arrays.

### Data acquisition and processing

Electroencephalographic (EEG) and EOG signals (0.01–100 Hz passband, 60 Hz notch filter) were digitized at 200 Hz. The EEG was recorded from 12 scalp sites (F3/Fz/F4, C3/Cz/C4, P3/Pz/P4, O1/Oz/O2) and the right mastoid. Scalp electrode sites were initially referenced to the left mastoid and re-referenced off-line to averaged mastoids. The vertical EOG was recorded from bipolar-referenced electrodes above and below the left eye. The horizontal EOG was recorded from bipolar electrodes at the outer canthi of the eyes.

**Figure 6:**
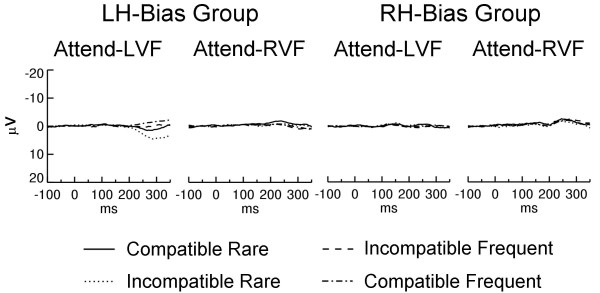
**Horizontal EOG data**. Grand average horizontal EOG data in each condition for the hemispheric utilization bias groups are shown. Note that stimulus offset was at 150 ms. In the recording procedures used, 1° of visual angle was equal to approximately +/- 20 μV EOG amplitude.

Trials were excluded from further analysis for the following reasons: 1) the horizontal EOG exceeded 1° of visual angle (calculated from calibration data obtained for each participant); 2) amplifier blocking occurred; and 3) no response was made by 1750 ms. After horizontal eye-movement rejection was performed (see results in Fig. 6), vertical EOG artifacts were removed from the EEG by an eye-movement correction method [43]. Averaged ERPs for each combination of attention condition and stimulus array were derived from correct response trials, collapsed over response hand, and baseline-corrected with a 100-ms prestimulus baseline.

### Data analysis

Behavioral performance measures were error rate and median RT. Errors were defined as incorrect target classifications (i.e., circle as square). ERP average amplitudes were measured at the electrode sites and latency windows where the components were maximal, based on the grand averages. ANOVAs consisted of the design: hemispheric utilization bias Group (LH-Bias/RH-Bias) × Attention condition (Attend-LVF/Attend-RVF) × Flanker (Compatible/Incompatible Rare) × Electrode site (for ERP amplitudes, Fz/Cz). ANOVAs were performed with the Greenhouse-Geisser correction reflected in the reported *p *values.

## Authors' contributions

KMS and MTB designed the study. KMS acquired and analyzed the data, and drafted the manuscript. MTB also helped to draft the manuscript.
